# The phase space of meaning model of psychopathology: A computer simulation modelling study

**DOI:** 10.1371/journal.pone.0249320

**Published:** 2021-04-26

**Authors:** Johann Roland Kleinbub, Alberto Testolin, Arianna Palmieri, Sergio Salvatore

**Affiliations:** 1 Department of Philosophy, Sociology, Education, and Applied Psychology, University of Padua, Padua, Italy; 2 Department of General Psychology, University of Padova, Padua, Italy; 3 Department of Information Engineering, University of Padova, Padua, Italy; 4 Padova Neuroscience Center, University of Padua, Padua, Italy; 5 Department of Dynamic and Clinical Psychology, and Health Studies, Sapienza Università di Roma, Rome, Italy; Fuzhou University, CHINA

## Abstract

**Introduction:**

The hypothesis of a general psychopathology factor that underpins all common forms of mental disorders has been gaining momentum in contemporary clinical research and is known as the *p* factor hypothesis. Recently, a semiotic, embodied, and psychoanalytic conceptualisation of the *p* factor has been proposed called the Harmonium Model, which provides a computational account of such a construct. This research tested the core tenet of the Harmonium model, which is the idea that psychopathology can be conceptualised as due to poorly-modulable cognitive processes, and modelled the concept of Phase Space of Meaning (PSM) at the computational level.

**Method:**

Two studies were performed, both based on a simulation design implementing a deep learning model, simulating a cognitive process: a classification task. The level of performance of the task was considered the simulated equivalent to the normality-psychopathology continuum, the dimensionality of the neural network’s internal computational dynamics being the simulated equivalent of the PSM’s dimensionality.

**Results:**

The neural networks’ level of performance was shown to be associated with the characteristics of the internal computational dynamics, assumed to be the simulated equivalent of poorly-modulable cognitive processes.

**Discussion:**

Findings supported the hypothesis. They showed that the neural network’s low performance was a matter of the combination of predicted characteristics of the neural networks’ internal computational dynamics. Implications, limitations, and further research directions are discussed.

## Introduction

Based on evidence of substantial correlation among psychopathological characteristics [1–4], several authors have recently proposed the hypothesis of a general psychopathology factor—the so-called *p* factor hypothesis—underpinning all common forms of mental disorders [5]. In analogy with the *g* factor [6, 7], explaining the positive correlation among all cognitive test scores, the *p* factor has been seen as a way to account for individuals’ propensity to develop any and all forms of psychopathological conditions.

Various studies have provided empirical support for the *p* factor hypothesis (e.g., [8–10]), showing that it predicts mental disorders and behavioural problems (e.g., difficulties in academic performance). In the last decade, the scientific literature has proposed a broad range of candidate constructs to represent the *p* factor, such as super-ordered personality trait [11], unpleasant affective state [12], low impulse control [13], deficit in cognitive function [14], and psychological liability [5]. These partially conflicting explanations seem to share, at their core, the same general approach, i.e., the idea that the *p* factor reflects a given single (or restricted set of) latent construct(s) to be detected empirically. The *p* factor hypothesis has drawn attention because of its potential to fulfil the need for a unified interpretative framework of psychopathology in the broader context of the ongoing debate on the validity of psychiatric/psychopathological diagnoses [15, 16].

There is still no agreement on its clinical significance or the underpinning mechanisms [17]. Recently, the *p* factor has been interpreted as the expression of the rigidity of meaning-making (i.e., a way of making sense of experience, characterised by low variability) [18, 19]. The idea that interferences in the way meaning-making develops represents a pathway for psychopathology that has been discussed by Tronick and Beeghly [20] in the context of infant research. The authors describe how infants’ mental growth is driven by so-called ‘acts of meaning’, which consist of the collection and organisation of information about their environment in superordinate dimensions. For instance, through their ongoing engagement with caregivers, infants create internal working models (i.e., new meanings) that can either be based on feelings of security or the need to defend themselves from some of these engagements, possibly leading to maladaptive behaviours later in life.

In accordance with this perspective, a semiotic, embodied, and psychoanalytic conceptualisation of the *p* factor has been proposed—the Harmonium Model [18]—which focuses on the global dynamics underpinning the meaning-making process. The specificity of the harmonium model lies in the fact that it provides a computational account of the *p* factor, and therefore of psychopathology; namely, a fine-grained depiction of the operative rules of the way it works.

### The harmonium model and the phase space of meaning

The Harmonium Model is grounded in the well-established general view of psychopathology as rigidity in meaning-making, the person’s tendency to interpret and respond in more or less the same ways to different self-environmental patterns. As a result, the meaning-maker is unable to take the relevant facets of the context into account to address the environmental demands successfully (e.g., [21, 22]).

**Fig 1:**
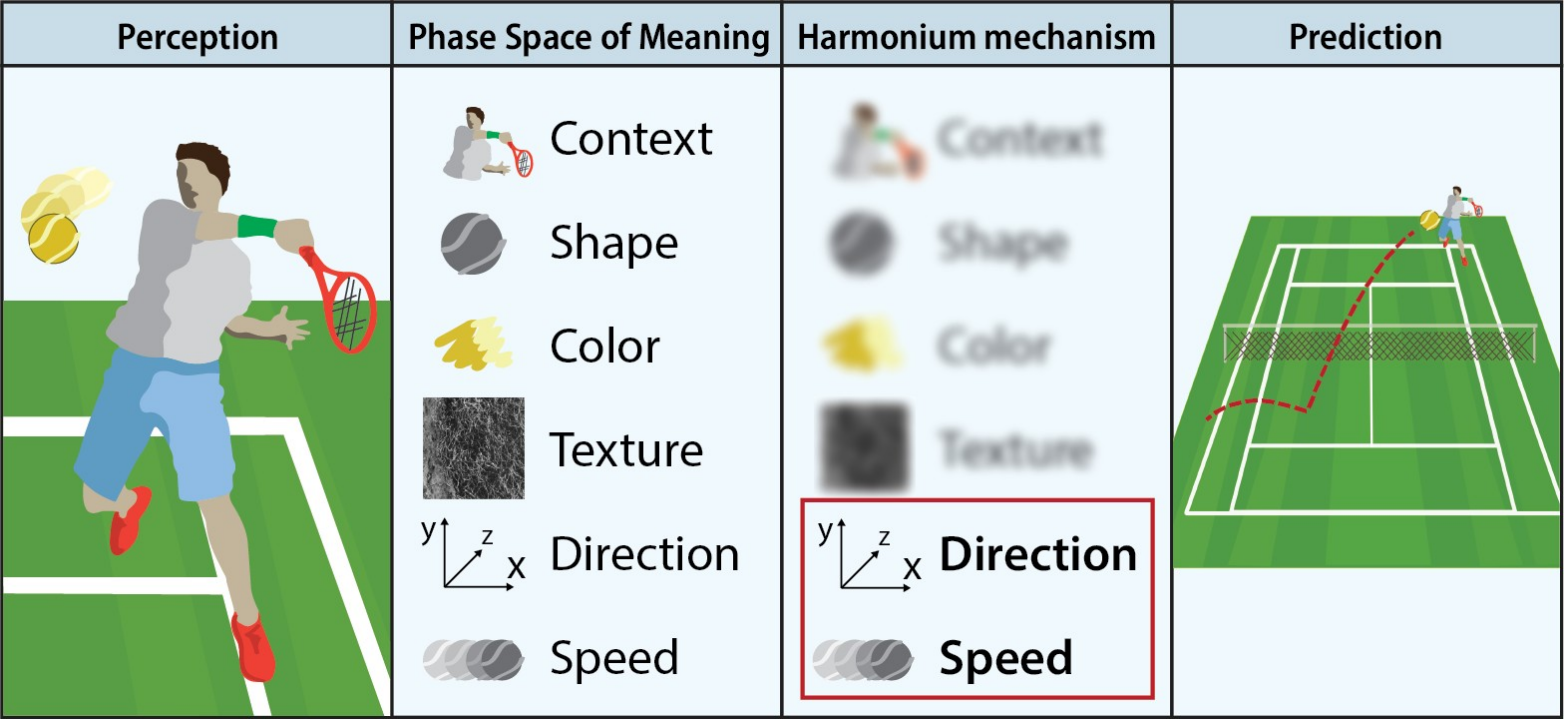
The harmonium model. The infographic represents a simplified application of the Harmonium Model, following the example in the text. A tennis player looking at their opponent’s serve is faced with a very complex perception. The phase space of meaning is a multidimensional space where any ‘object’ of perception (in this example, the ball) is represented in a broad range of dimensions. Not all these dimensions are needed to achieve a precise forecast of the ball’s trajectory, and thus inform future action (where to move to return the serve). The harmonium mechanism operates a dimensionality reduction in order to focus cognitive resources on the dimensions crucial to the task at hand.

The Harmonium Model (schematically illustrated in Fig 1) provides a model of rigidity of meaning-making underpinning psychopathology. It can be described in terms of one major assumption, one related corollary, and four tenets (for a systematic presentation, see [18]).

#### Assumption: Cognition is at the service of action

According to Embodied Cognition, cognition works in terms of online dynamic sensorimotor representations of the environment, shaped by the need to coordinate the person’s interaction with it [23–26]. The general rule and ultimate purpose of cognition is to keep the action coupled with ever-changing environmental states.

#### Corollary: Cognition is future oriented and searches for fit

In order to keep action coupled with a continuously changing environment, cognition is oriented to forecast the incoming variation of the environmental state [27, 28]. More particularly, cognition uses the information from sensory input to forecast the incoming environmental state and complements the forecast with the simulation of a response that enables the fit of the forecast to be upheld, that is, the coupling between action and environment.

#### Tenet 1. The Phase Space of Meaning (PSM)

Cognition can be modelled as an ongoing activity of mapping the moment-by-moment environmental states in terms of dimensions of meaning, each of which maps a component of the environmental variability. The *Phase Space of* Meaning (PSM) model is the representational space defined by these dimensions. For instance, a ball can be represented as a point on the PSM having as coordinates the values on dimensions like form, colour, function, owner, and so forth.

#### Tenet 2. PSM dimensionality modulation

Given that at any moment most of the environment’s characteristics are not relevant for the regulation of action, the cognitive system has to background most of the environmental complexity, and in so doing, bring only the pertinent facets to the fore [29]. For instance, the tennis player is interested in the speed and direction of the ball, not its colour. This process of backgrounding/foregrounding can be modelled in terms of the modulation of the PSM dimensionality: the cognitive system reduces potentially infinite PSM dimensionality by giving salience to a subset of dimensions only. In this way, the cognitive system selects a portion of the environmental variability to be processed, sourced from environmental components mapped by the active PSM dimensions.

#### Tenet 3. The harmonium mechanism

The modulation of PSM dimensionality is a way of optimising the trade-off between capacity of fit and the information power of the forecast. Indeed, the lower the dimensionality, the fewer the components of environmental variability taken into account; therefore, the lower the uncertainty the forecast has to address. In the final analysis, this means that the forecast prioritises the capacity of fit (i.e., its success) over its information power (i.e., its capacity of reducing uncertainty). The greater the dimensionality, the more the uncertainty, and the higher the forecast’s information power. In summary, like a harmonium, the cognitive system expands and contracts its inner space moment by moment in order to optimise the balance between the reproduction of its inner organisation (i.e., the capacity of fit) and the need for action regulation (i.e., the information power) [18].

#### Tenet 4. Psychopathology and poor modulation of PSM

The rigidity of meaning-making, which is typical of psychopathology, can be modelled in terms of a poorly modulable PSM. The Harmonium Model holds that PSM is characterised by two different kinds of dimensions [18] (see also [23]). *Primary dimensions*, namely a few basic dimensions corresponding to generalised, embodied affect-laden meanings (e.g., pleasantness vs unpleasantness) are the basis of meaning-making and provide global, homogenising connotations of the field of experience as a whole [30]. Due to these characteristics, the primary dimensions tend to be invariant within and between individuals [31, 32]. Thus, they constitute the stable nucleus of the PSM: dimensions that are activated in any meaning-making process, regardless of the specificity of the environmental states (for a model of affects consistent with this view, see [33]). *Secondary dimensions*, each of which corresponds to a property/quality through which a certain set of environmental states (e.g., an object, a circumstance), can be represented. A car might be represented as being speedy, expensive, or safe, but not as tasty, happy, or lazy. The secondary PSM dimensions correspond to information-oriented meanings, activated variably by the characteristics of the environmental states and, more generally, the need for action regulation. The modulation of the PSM concerns the secondary dimensions. The Harmonium Model maintains that the higher the weight of these dimensions within the PSM, the more it is capable of modulation; conversely, a poorly modulable PSM reflects the high weight of primary dimensions and the low weight of secondary dimensions. The high weight of primary dimensions means that most of the meaning-making is saturated by the invariant affective meanings (e.g., the connotation of experience in terms of friend-foe affect-laden schema; see [34]), while little room is left for further PSM components, namely, the components on which the detection of the nuances of the experience depends [23]. In brief, the high weight of the primary dimensions can be considered the computational model of a cognitive system constrained to provide simplified, homogenising interpretations of the environment, unable to attune its variability, and therefore to address the requirements of action regulation.

In what follows, a clinical vignette is used to exemplify the view of psychopathology in terms of dimensionality. James is a 65-year-old patient, meeting DSM-5 criteria for narcissistic personality disorder, followed in individual psychotherapy by one of the authors. He addresses any circumstance and events, both in interpersonal and job contexts, in terms of highly salient generalised affective connotation; in this case, as if it were destined to lead to a defeat and/or a negative evaluation by a severe judge. Sometimes, he feels he is lucky to avoid failure. Most of his feelings and actions are modelled by the anticipation of or reaction to this dichotomy: failure vs. avoiding failure. This dichotomy constitutes the major affect-laden meaning (i.e., the primary dimensions of James’ PSM), in terms of which the patient maps most of the variability of his environments, and by which he represents circumstances and their evolution, with little room left for secondary dimensions. As a result, the patient is rigidly enslaved to an invariant mode of meaning-making, unable to detect important environmental facets and therefore modulate his feelings and actions accordingly. Such facets affect his responses to his significant others’ desire towards him and others, his own and their needs and plans, and so forth. Such an inability considerably narrows his capacity to adjust, e.g., learn from feedback and enter reciprocal, symmetrical relationships, leading him to assume the position of one who has to protect himself from the risk of failure, regardless of the actual content of the circumstances.

### Aims and hypothesis

The Harmonium Model of psychopathology is in its first stage of development. Thus far, it is supported by clinical and theoretical justifications and indirect empirical evidence only (see [18] for a review). For instance, Tonti and Salvatore [35] found that the dimensionality of meaning-making is directly associated with markers of emotional activation and affect-laden forms of evaluation. Ciavolino and colleagues [36] developed a method of estimating interrater reliability based on a measure related to the dimensionality of judgment. Salvatore and colleagues [37] showed that affect-laden, polarised, identity-oriented evaluations and attitudes towards relevant social issues (e.g., foreigners) are associated with low-dimensional worldviews.

This indirect evidence is encouraging but far from conclusive. The purpose of this simulation study is to move a step ahead in the development of the PSM model by providing a first direct test of the core tenet of the Harmonium Model, the idea that psychopathology can be modelled at a computational level as a low-dimension PSM. Our study adopts a simulation design, based on a deep learning model [38] simulating a cognitive process, in this case, a classification task. It considers the level of task performance as the simulated equivalent of the cognitive process, and therefore its position on an ideal normality-psychopathology continuum. Moreover, it considers the dimensionality of the neural network’s internal computational dynamics (ICD) as the simulated equivalent of the PSM’s dimensionality. Our computational approach is based on a popular cognitive architecture that relies on energy-based models [39], which can simulate a form of ‘learning by observation’ that does not require labelled examples and exploits Hebbian learning principles [40]. In line with predictive coding theories of the brain [28, 41], learning in these models corresponds to fitting a hierarchical probabilistic generative model to the sensory environment. A separate decision layer was implemented as a read-out classifier, which was stacked on top of the deep network in order to simulate an explicit behavioural task [42–44].

On this basis, this study is aimed at testing the following hypothesis: The neural network’s level of performance is positively associated with the dimensionality of its internal computational dynamics. More specifically, it is expected that: (a) the higher the weight of the primary dimensions and (b) the lower the weight of secondary dimensions, the lower the performance will be. Incidentally, it must be noted that (a) and (b) are worth considering separately because, though the primary and secondary dimensions are complementary within each individual PSM, the magnitude of both vary between PSMs (i.e., a certain PSM can be characterised by a higher or lower weight of both primary and secondary dimensions than another PSM). Despite our modelling approach being inspired by recent advances in artificial neural networks and deep learning research, our goal here is not to implement a state-of-the-art machine vision system, but rather to operationalise a recent psychopathological theory in the form of a computational model.

## Materials and methods

### Design

Two studies were performed.

#### Study 1

In order to generate variability in the neural networks’ performance, two instances of the same neural network architecture were subjected to different training conditions (TC): high entropy (TC-HE) and low entropy (TC-LE). After an unsupervised learning phase, the two instances were tested on a classification task, which required reading out the internal representations of the stimuli by mapping them into one of the 26 possible categories (see details below). Finally, the relationship between classification performance and dimensionality of the internal computational dynamics was estimated.

#### Study 2

This second study was carried out to collect sample statistics that allowed controlling for the possible effect of the idiosyncratic characteristics of the single instances used in Study 1. To this end, Study 2 replicated the procedure and analyses of Study 1 on a set of 40 replicas (20 each subjected to TC-HE and 20 to TC-LE) of the same neural network architecture. To reduce the computational complexity of the simulations, analyses were performed on only two categories of stimuli.

### Materials and procedures

**Fig 2:**
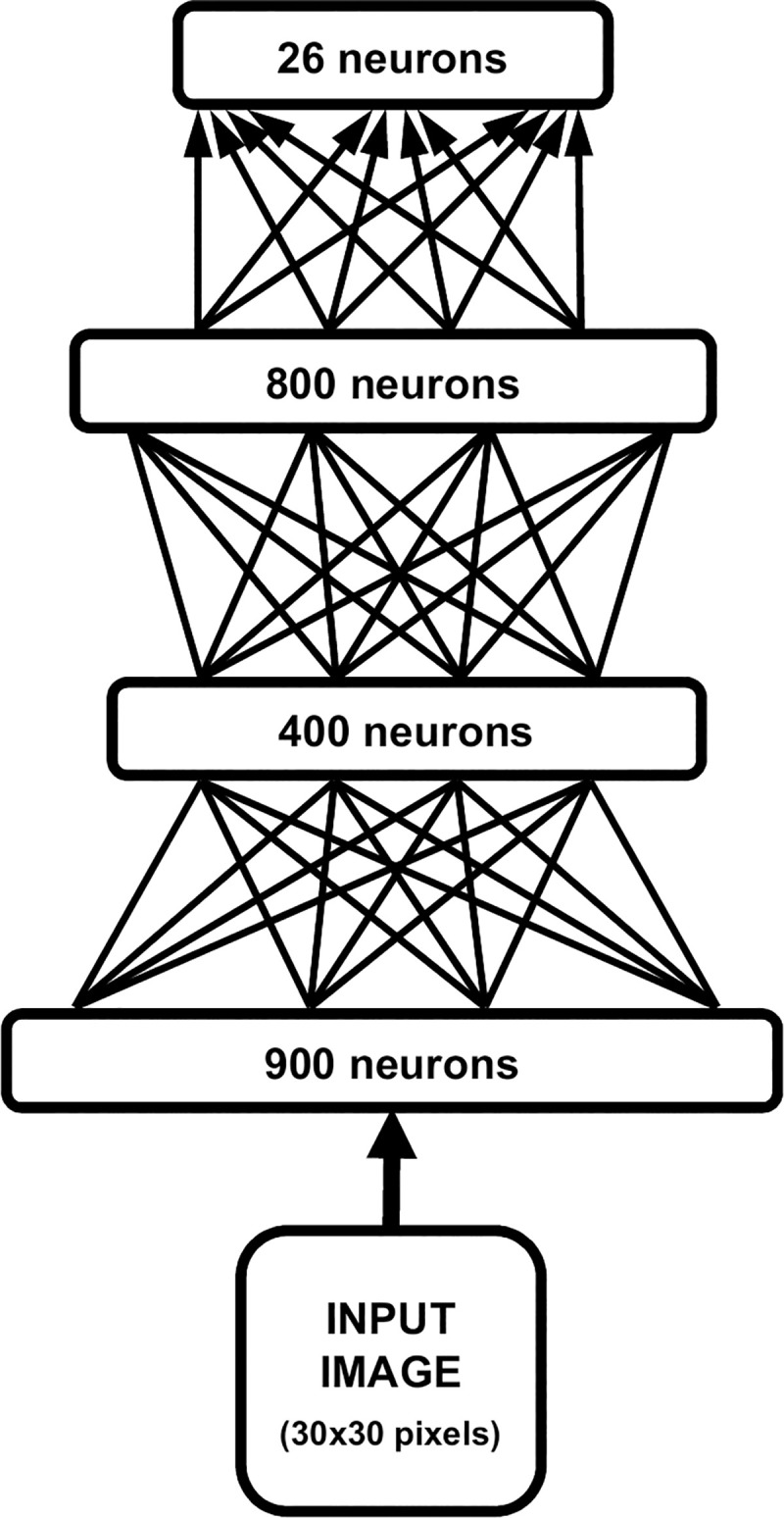
Architecture of the deep learning model. The input image was vectorised and presented through an input layer (900 neurons). Two hidden layers of 400 and 800 neurons, respectively, were then used to learn a hierarchical generative model in a completely unsupervised fashion (undirected arrows). The behavioural task is finally simulated by stacking a read-out classifier on top of the network (directed arrows).

Both studies relied on a deep learning model consisting of a multi-layered deep belief network [39]. This kind of neural network is able to learn hierarchical representations by progressively discovering more abstract features from sensory input [45]. Our model is based on a recently proposed architecture that has been used to simulate the psychophysics of letter recognition in humans [42]. Fig 2 describes the neural network’s architecture. It comprises: a) one input layer (900 neurons), which receives the vectorised stimulus encoded as grey-level activations of image pixels; b) two layers of hidden neurons (400 and 800 neurons, respectively), deployed to learn a set of visual features from the sensory data [46]; and c) one output layer (26 neurons) that is used to simulate the behavioural response—each output neuron corresponds to one of the possible perception classes (i.e., letters). During the unsupervised learning phase, the network extracted a set of visual features by learning a generative model of its environment (i.e., the training dataset). Connection weights were adjusted using the contrastive divergence algorithm, which implemented a Hebbian-like learning rule [47]. Learning continued for 40 epochs, which guaranteed convergence of the reconstruction error. In order to simulate a behavioural response, a supervised phase was then carried out by training a read-out classifier on the top-layer internal representations of the model (for details, see [46]). Connection weights were adjusted using the delta rule, which implemented a simple form of associative learning.

**Fig 3:**
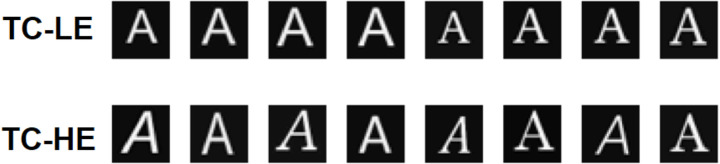
Samples of visual stimuli. Images from the low entropy dataset (TC-LE) contain letters printed using a limited number of variability factors, while images from the high entropy dataset (TC-HE) were created using a greater variety of fonts and styles.

In both studies, neural networks were attributed to one of the two training conditions (TC-HE and TC-LE), each of which was characterised by a set of training inputs. TC-HE and TC-LE sets were designed to have the same number of stimuli (26 letters) and the same global amount of information, but a different entropy, namely a different degree of within-set variability: high and low entropy, in the TC-HE and TC-LE, respectively. In the case of the TC-HE, the set of stimuli consisted of 27 different versions of each of the 26 letters, obtained by the combination of nine fonts and three styles (normal, italic, and bold). In the case of the TC-LE, the set of stimuli consisted of two versions of each of the 26 letters (two fonts, no style variation). In order to equalise the number of learning trials used in the two training conditions, the TC-LE stimuli were replicated several times until the size of the two training sets were matched. Fig 3 reports some instances of the set of inputs used.

In Study 1, after the unsupervised learning stage, the two instances of neural networks were applied to the same classification task. To this aim, a read-out classifier was stacked on top of each deep network and trained on the corresponding dataset used during the unsupervised phase. The classification accuracy was then evaluated on an independent test set consisting of letter images created using four new fonts and three styles (normal, italic, and bold), which were also reduced in contrast by dividing the pixel luminosity by a factor of two to make recognition more challenging.

In Study 2, we tested 40 instances of the neural network architecture (20 subjected to TC-HE and 20 to TC-LE) to check the possible effect of the specificity of the neural networks used in Study 1. Due to computational limitations, the analysis of the internal computational dynamics was performed only on two randomly sampled images of two letters (‘A’ and ‘M’).

### Measures

In order to check the design assumption that the TC-HE set of training inputs did not have a higher global amount of information than the TC-LE set, the number of active pixels characterising each training input set was calculated. To check the second design assumption—i.e., that the sets of training stimuli adopted in TC-HE had a higher level of entropy than the TC-LE set—the entropy was estimated in terms of dimensionality of the factorial space obtained by the principal component analysis (PCA), applied to the distributions of pixels. The higher the number of dimensions needed to explain the whole distribution, the higher the entropy (for a discussion of this approach, see [48]).

To control the effect of the complexity of the input signal to be classified, the latter was estimated by means of the perimetric complexity (PeCo). PeCo has been computed as the ratio between the surface and perimetry of the stimulus [49].

**Fig 4:**
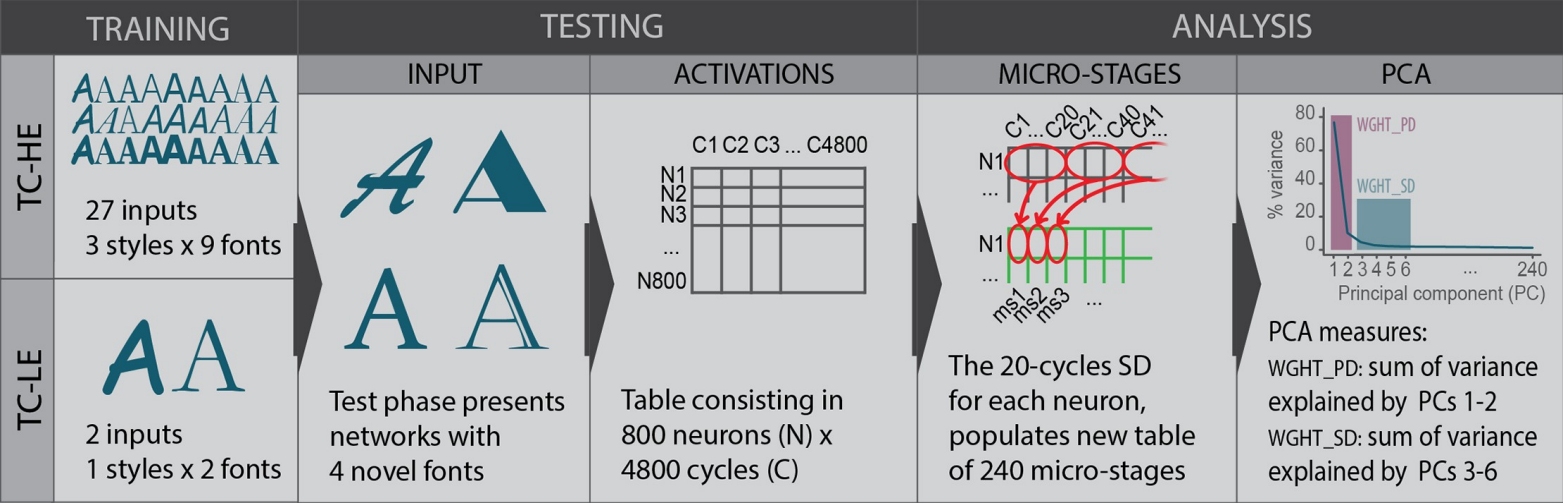
Schematic representation of the analytic procedures employed in the study. The diagram exemplifies the procedures of one TC-LE and one TC-HE network on the ‘A’ letter set of inputs. The same steps were repeated for all other letters and networks. Note that the figure’s fonts are exaggeratedly different in order to better convey the study design. For an example of the actual fonts employed, refer to Fig 3. PCs = principal components.

The performance of each classification task was measured as a percentage of correct classification. The neural network’s dimensionality of internal computational dynamics (ICD) was measured in terms of dimensionality of the factorial space obtained by the PCA applied to the dataset containing the activations of the hidden neurons recorded for each stimulus presentation during the unsupervised learning phase. More particularly, the following procedure was followed (see Fig 4).

First, the analysis focused on the 800 neurons of the second hidden layer, which interfaced the output layer, corresponding to the crucial stage of processing, where the information was subjected to abstraction and synthesis [46]. We considered the cycle activation of these neurons; that is, after every 120 learning events repeated for 40 epochs, we presented all letter images to the network, and we recorded the corresponding activation of every neuron in the second hidden layer. As a result, for each class of stimuli (i.e., for each letter), an 800 neurons*4,800 cycles matrix was built, with the *ij* cell reporting the degree of activation of the *i-th* neuron in the *j-th* cycle.

Second, given our focus on the neural network micro-dynamics, the local variation across close cycles was foregrounded. To this end, the standard deviation (SD) was calculated for each window of 20 adjacent cycles (one micro-stage). Thus, for each neuron 240 (4,800/20) micro-stage scores were calculated, each of them indicative of the variation of the neuron’s activation within 20 cycles. This led to building a second kind of matrix– 800 neurons*240 micro-stages.

This procedure was carried out separately for each neural network and each letter. This means that 132 (800*240) matrixes were built: 52 (26 letters*2 neural network) in Study 1, and 80 (2 letters*40 neural networks) in Study 2.

**Fig 5:**
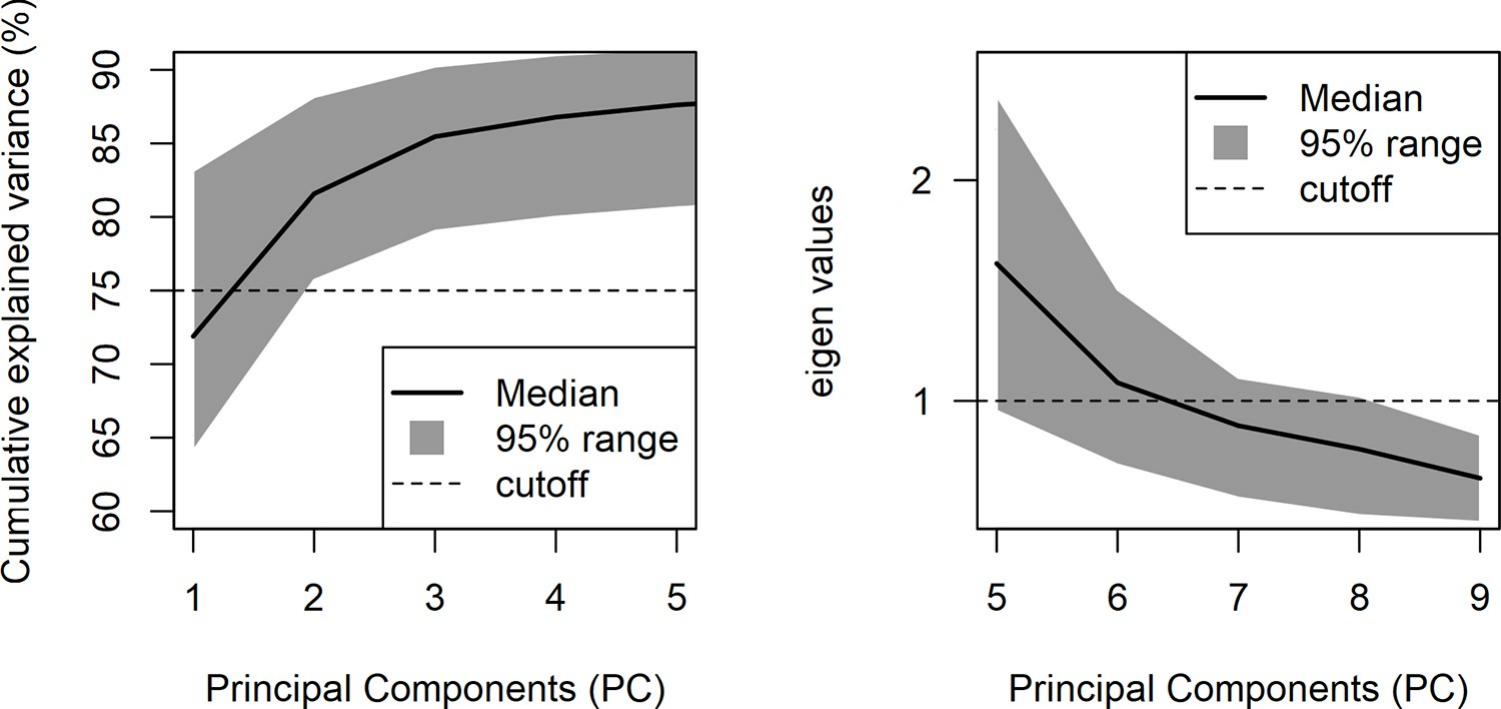
PCA distributions. The panels explain the decisional process employed to choose the number of components in WGHT_PD and WGHT_SD, based on the data of Study 1. Specifically, the left panel shows the distribution (median and 95% range) of the cumulative explained variance of the first five PCs across all PCAs. The right panel is a scree-plot representing PCs 5 to 9 focusing on the eigenvalue = 1 cut-off.

Each of these 132 matrixes was subjected to PCA separately. The dimensionality of the factorial spaces was analysed in terms of the following two indicators:

- *Weight of the primary dimensions (WGHT_PD)*. This indicator is the sum of the variance explained by the first two factors of each PCA. The choice to focus on such a number of factors is based on the preliminary analysis of the distribution of the variance across the PCAs, which showed that these components worked systematically as the main dimensions in PCA outputs. In 95% of the PCAs (5% were excluded as outliers), the first and second factors together explained no less than 75.71%, and the cumulative explained variance did not increase greatly once the third factor was added (from at least 75.71% to at least 79.06%), as can be seen in Fig 5, left panel. Additionally, the choice to consider the first two factors is consistent with the literature, which converges in detecting two basic dimensions of affective meanings, e.g., pleasure/displeasure and activated/deactivated [50, 51], or evaluation and dynamism [31]).
- *Weight of the secondary dimensions (WGHT_SD)*. This indicator is the sum of the variance explained by the relevant factors beyond the first two. In order to identify the relevant factors, a Kaiser-like criterion was adopted: the median number of factors having eigenvalues higher than 1 was calculated. The computation was performed separately for Study 1 and Study 2’s sets of PCAs; however, in both studies, factor 6 was identified as the last factor to have an eigenvalue higher than 1 in at least 50% of the PCAs, as represented in Fig 5, right panel. *WGHT_SD* was calculated as the sum of the explained variance from factor 3 up to factor 6.

### Data analysis

#### Preliminary check

1) The amount of information of the two training input sets was checked by means of a one-tailed Welch t-test. 2) The assumed different entropy between TC-HE and TC-LE’s training input sets was checked by means of visual inspection of the slope mapping the percentage of variance explained by the factorial component obtained by the two PCAs, applied separately to the distribution of active pixels in the two training input sets. 3) The assumed effect of the two training conditions in differentiating the neural networks’ performance was checked by means of a two-tailed Welch t-test with paired samples.

#### Studies 1 and 2

The relationship between the neural network’s performance and the dimensionality of its dynamics (ICD) was modelled by means of two multiple regression models. The networks’ performance was the dependent variable, and the characteristics of their dimensionality (WGHT_PD and WGHT_SD) and the letters’ perimetric complexity were the independent variables. All independent variables were transformed to standard scores. Although the two parameters of dimensionality (WGHT_PD and WGHT_SD) are related to each other as part of a global distribution with a sum equal to 1, they are both worthy of being included as separate predictors, because each refers to a specific characteristic of the factorial space (i.e., the weight of primary and secondary dimensions), having at least a partial independent chance to vary with respect to the whole distribution of the variance across the factorial dimensions. This is shown by the fact that, taken together, WGHT_PD and WGHT_SD refer to the first six of the 240 factors extracted by each PCA. Finally, each letter is characterised by a specific PeCo, so the introduction of the PeCo in the regression models operates as a control for the possible letter effect.

All analyses were performed using *R* statistical software, version 3.6.3 [52], and PCAs were performed with the *Psych* package, version 1.9.12 [53].

## Results

### Preliminary checks

#### 1) Amount of information

In order to verify that the amount of information in the TC-HE training set was not higher than TC-LE, a one-tailed Welch t-test was performed on the number of active pixels characterising each training input set (*M*_TC-LE_ *=* 102.96, *SD*_TC-LE_ = 27.99; *M*_TC-HE_ = 93.82, *SD*_TC-HE_ = 33.42). The test showed no significant evidence to reject the null hypotheses (*M*_TC-LE_−*M*_TC-HE_< 0), *t*(81712) = 43.05, *p* = 1.

#### 2) Comparison of the entropy of the training input sets

**Fig 6:**
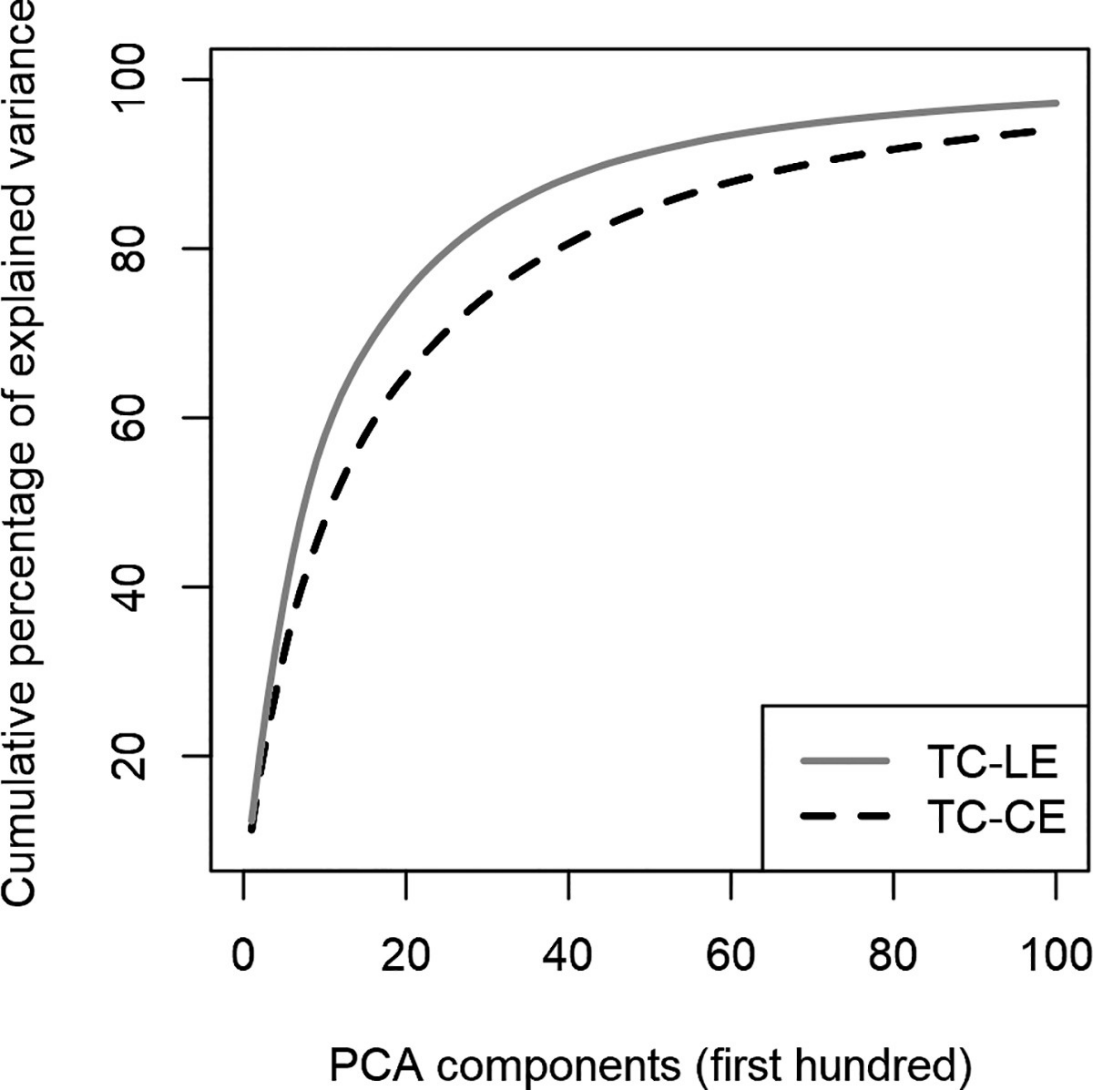
Comparison between TC-LE and TC-CE PCA distributions. The lines represent the cumulative percentage of explained variance for the first 100 components of TC-LE and TC-CE networks. A lower explained variance per factor implies a higher entropy.

Fig 6 compares the distribution of the percentage of total variance explained by each factorial dimension obtained by the two PCAs. Each of them is applied to the active pixel distribution of one training input set (TC-HE *vs* TC-LE). As expected, the TC-HE is demonstrated to have higher entropy, as shown by the fact that more components are needed to explain the variance than in the TC-LE distribution. Though differences are not highly marked (the main difference concerns the last 1% of variance), they are consistent with the expected effect pursued by the design. Indeed, even if the two training input sets were designed with different degrees of complexity, the visual structure of the inputs used on the two sets are mostly similar to each other.

#### 3) Effect of the training condition

The TC-HE network (*M* = 0.913, *SD* = 0.107) achieved a higher performance than the TC-LE network (*M* = 0.475, *SD* = 0.220) for each of the 26 letters of Study 1. A Welch two-sample t-test with paired samples was significant, with *t*(25) = 12.87, *p* < 0.001. This result shows that the limited variability of the TC-LE state resulted in overfitting the training data, preventing a flexible generalisation of the learned features to the test stimuli.

### Study 1

The regression model showed that performance decreased by -0.13 ± 0.037 (p < 0.0009) for each SD of WGHT_PD and increased by 0.073 ± 0.036 (p = 0.0465) for WGHT_SD. No association was found between perimetric complexity and performance (*ß*_PeCo_ = 0.056 ± 0.037, p = 0.1288). The model’s intercept (representing performance values expected with average dimensionality and letter complexity) was estimated at 0.694 ± 0.035. The model had an adjusted R^2^ of 19.86%, showing a moderate fit.

### Study 2

The same model was used for Study 2 datasets, except for the perimetric complexity that was not included, given that only two letters were considered in this case, and on the basis that PeCo showed no effect in Study 1. The model estimated similar parameters to Study 1’s with higher precision, given the increased sample size. Specifically, dimensionality predicted lower performance levels by -0.24 ± 0.007 (*p* < 0.0001) for WGHT_PD, and higher performance levels by 0.618 ± 0.007 (*p* < 0.0001) for WGHT_SD. The model’s intercept (representing performance values expected with average dimensionality and letter complexity) was estimated at 0.618 ± 0.007. The model had an adjusted R^2^ of 94.59%, showing a high fit.

## Discussion and conclusion

Study 1 and Study 2 regression models supported the hypothesis. As expected, the neural networks’ level of performance was negatively associated with WGHT_PD and positively associated with WGHT_PD. This showed that low performance was determined by the combination of the high weight of primary dimensions (WGHT_PD) and low weight of secondary dimensions (WGHT_SD) of the neural networks’ internal computational dynamics.

Preliminary checks and statistical controls ensured that these results were indicative of the hypothesised relation between dimensionality and performance.

- The three assumptions underlying the studies’ procedure were verified. Although the TC-HE training input set was not characterised by a higher amount of information than the TC-LE set, the network trained over the complex dataset presented higher entropy. As expected, the two training conditions were effective in differentiating the levels of neural network performance.
- Study 1’s regression model showed no residual effect of the letters’ perimetric complexity on the networks’ performance. This leads us to conclude that the effects detected by the regression model were independent from the variability related to inherent characteristics of input letters.
- Study 2’s regression model replicated the Study 1 regression model over a larger sample of trained models. This indicates that the performance-dimensionality relation was not due to the idiosyncratic characteristics of the Study 1 neural networks. On the contrary, the larger magnitude of the effect of dimensionality on performance shown by Study 2 suggests that, if the specific characteristics of Study 1 neural network had played a role, it would have been in the direction of underestimation of the effect of dimensionality on performance.

In summary, if we assume that the relationship between neural networks’ performance and dimensionality of its internal computational dynamics simulates the relationship between psychopathology and PSM dimensionality, the main findings of Studies 1 and 2 are consistent with the Harmonium Model interpretation of the *p* factor, namely that psychopathology is due to a poorly modulable Phase Space of Meaning (PSM) as a consequence of a combination of high incidence of affect-laden meanings and low incidence of information-oriented meanings [18].

Some implications of our simulation model are worth highlighting. First, to the best of our knowledge, the two studies reported in the present paper are the first attempts to adopt a simulation framework based on state-of-the-art computational cognitive models in the analysis of psychopathology. Connectionist models, network analyses, and artificial intelligence (AI) techniques (in which deep learning has its roots) have been used in clinical psychology research since the 1990s [54–62]. Yet, they are employed as data analysis tools to model the clinical phenomena under investigation, rather than to simulate the cognitive processes underpinning psychopathology (a partial exception is provided by Del Giudice [10], which simulated life trajectories in samples of virtual psychiatric patients). With respect to these studies, the current paper shows that the simulation approach can provide an efficacious way to build formal models of the computational mechanisms underpinning clinical phenomena, and in so doing, contribute to opening the black box of psychopathology [18, 62].

Second, it is worth mentioning that the factorial spaces used to represent the neural network’s internal computational dynamics were demonstrated to have an inner organisation consistent with the Harmonium Model’s view of the PSM as characterised by an affect-laden nucleus comprised of two (or very few) basic dimensions [23]. Indeed, the preliminary analysis of the distribution of the explained variance showed that the first two factorial dimensions covered ¾ of the whole variance in at least 95% of the 132 PCAs performed. This finding may not be considered a direct support for the Harmonium Model’s assumption (and indeed it was not included in the hypothesis), but it should be considered a computational characteristic of the simulation model, which, due to its consistency with the simulated process, supports its construct validity.

Third, the relationship between the entropy associated with the two training conditions and performativity is worth discussing. This relationship is not contained in the paper’s hypothesis; indeed, the low vs high entropy training conditions were implemented instrumentally to obtain the required variability of the neural networks’ performance. However, this does not prevent us from pointing out that the relation between entropy/complexity and performance is analogically evocative of an ontogenetic model of psychopathology, namely, of the idea that the PSM low-modulation underpinning psychopathology is due to the low complexity of the ontogenetic environment. According to this view, PSM rigidity at the basis of psychopathology is the consequence of a flow of past environmental states that has been unable to ‘challenge’ the cognitive system, and therefore compel it to develop its dimensionality to map the environmental complexity. That being said, although studies’ findings do not disconfirm this idea, they do not support it either. It is not possible to discard the alternative hypothesis that the different complexity of the neural networks’ internal computational dynamics is due to other causes (e.g., the genetic equipment), which the training conditions have set in the neural network, without being their computational analogue. The analogy is clear and quite provocative, thus motivating further exploration.

Fourth, the method we introduced for analysing the activation dynamics of hidden neurons might also be successfully applied in other contexts, for example, to quantitatively characterise the representational capacity of deep neural networks. In the field of machine learning, this topic is currently being investigated by a variety of approaches based on dimensionality reduction [63], trajectory analysis [64], algebraic topology [65], and manifold theory [66], among others. Furthermore, our approach, based on the eigenspectrum analysis of the internal activations’ dimensionality, might potentially be combined with graph theoretical measures to provide further insights into the relationship between structural and functional properties of neural networks [67]. Very recent AI research has shown that the empirical spectral density of deep networks’ weight matrixes can be used to characterise the self-regularisation properties of these models [68], and that spectral properties of the weight matrixes (such as power-law fits of eigenvalues distribution) can provide useful metrics to assess the quality of deep learning models [69]. Others have shown that the effective dimensionality of the parameter space of deep networks can be estimated by computing the eigenspectrum of the Hessian on the training loss, providing a useful proxy to characterise model complexity and generalisation performance [70]. These results suggest that eigenspectrum analysis can be successfully applied to study the inner organisation of deep networks at the connectivity level, providing a useful complementary perspective to the activation-based analysis we introduced here.

From a broader perspective, the present study aims to offer a contribution to the identification of a diagnostic strategy characterised by a limited number of parameters, addressed to satisfy the increasing need for the validity of psychopathological diagnoses [15, 16]. The conception of psychopathology, also in terms of an embodied, evidence-based factor, could lead to overcoming some impasses of the current nosographic/tassonomic classification systems, which are heterogenous and not scientifically falsifiable [71], contributing to a more effective diagnostic process and, consequently, more appropriate therapeutic strategies.

Before concluding, limitations of the studies have to be highlighted to recognise the constraints on the generalisability of the findings. Due to their pioneering nature, the two studies reported in the current paper are based on only one neural network architecture, one kind of cognitive function (a classification task), one set of input stimuli (letters), and two training conditions.

Finally, a general caveat is worth mentioning. The findings discussed above have to be interpreted as supporting the hypothesis that psychopathology can be modelled at the computational level in terms of dimensionality of the cognitive system’s functioning. This is a necessary but insufficient condition to conclude that psychopathology actually works in this way. On the other hand, the hypothesis focused on by the current simulation study was not intended in ontological terms as explicative of the actual mechanism of psychopathology. Rather, the aim of our simulation study was to test the Harmonium Model as a valid computational model, able to describe the computational micro-dynamics assumed to underpin psychopathology. The support obtained for this hypothesis paves the way for further studies, conveying more complex and realistic conditions, and aimed at developing further the phase space of meaning model and using it to support the explicative mechanisms of psychopathology suggested by the Harmonium Model.

More precisely, subsequent studies could increase—qualitatively and quantitatively—the gamut of neural network architectures, tasks, input sets, and training conditions. For example, a promising venue for future research would be to scale up our model to more realistic stimuli, such as images of faces [72] and real objects [73]. This would require adopting more advanced deep learning approaches, such as modern computer vision models based on convolutional architectures [74]. Moreover, specifications and constraints on the neural network models will need to be introduced, and reverse engineering procedures carried out. This would increase the validity of the simulation model with respect to the clinical phenomena they refer to. In doing so, we intend to go ahead in the Harmonium computational modelling of psychopathological meaning-making and of the role of affects in it.
