# Supplementary material for: The phase space of meaning model of psychopathology: A computer simulation modelling study
Source: PLoS One. 2021 Apr 26;16(4):e0249320. doi: 10.1371/journal.pone.0249320 (PMC8075201; doi:10.1371/journal.pone.0249320)
Supplement: Attachment — Submitted filename: response to Reviewers_Harmonium_NeuralNet_2021-01-17.docx [file pone.0249320.s001.docx]

Dear Professor Chi-Hua Chen,

Academic Editor of Plos One journal,

I am presently submitting a revision of the paper re-entitled, according to reviewers suggestion: ‘The Phase Space of Meaning Model of Psychopathology. A Computer Simulation Modeling Study”, by Alberto Testolin, Arianna Palmieri^,^ Sergio Salvatore and me.

As requested by PLOS One Policy, a marked-up copy of the manuscript that highlights changes made to the original version labeled 'Revised Manuscript with Track Changes' has been uploaded. Namely, edits suggested by Reviewer 1 has been highlighted in green; edits suggested by Reviewer 2 are highlighted in yellow, including the new references. An unmarked version of the revised draft without tracked changes has been upload as a separate file labeled 'Manuscript'.

The minimal data set can be retrieved now at the following link: https://osf.io/96vhp/

We are indebted to you and the reviewers for the attention devoted to our work, and we hope you will appreciate the revised version of the paper and find it worthy of publication on your journal.

Best regards

Johann R. Kleinbub

**Reviewers Responses**

Reviewer #1:

This paper examines in-depth the fundamental issue of the continuum between normality and psychopathology by use and implementation of computational models, including neural networks/computer simulation techniques. Authors manage to defend the view that there exists a dimensional model of psychopathology, anchored in the Phase Space of Meaning concept developed on the level of computational modelling. The paper will benefit from few amendments:

1. Deliver more precise and straigtforward conclusion in terms of core message to more general mental health audience without background in cognitive science. Perhaps a summary of main contributions would fit the purpose.

*A core message to more general mental health audience without background in cognitive science has been added, in a short summary, at the end of Discussion section, as indicated by the reviewer. In this vein, the following reference has been* added: (Stoyanov, 2020)

2. Include some critical premises from methodological perspective, which are relevant to that core message, e.g.
<https://link.springer.com/content/pdf/10.1007/s13148-010-0014-2.pdf>
<https://www.frontiersin.org/articles/10.3389/fpsyt.2019.00869/full>

*We thank the reviewer for the suggested literature. We included the suggested citations also in the short summary requested above* (Stojanov et al., 2011; Stoyanov et al., 2019)*.*

Reviewer #2:

The authors seek to prove that the Harmonium model can be used to explain general psychopathology. Their theory states that rigidity in meaning-making is psychopathology, which can be represented through the Phase Space of Meaning (PSM).
They hypothesize that a PSM that puts more emphasis on its primary dimensions (representing the global meaning of the environment exposed too) and rather than its secondary dimensions (representing minor subjective details of the environment) leads to psychopathology.

They test this by learning a classification network and looking at the network's inner layers through training. They conceptualize the change in primary dimensions as the standard deviation of the first two PCs (wght_pd) and the change in secondary dimensions as the standard deviation of the other PCs (wght_sd) (with eigenvalue higher than 1). They find that high SD for wght_sd means higher accuracy, while the opposite is true for wght_pd. Thus, they conclude that this reflects how focusing only on the primary dimensions leads to worse internal decisions (which means psychopathology from their theory).

1) The Harmonium model's description is quite complicated; I understand that compressing the description into a few pages is difficult. What would really help is some infographics representing the PSM, the ongoing signal, the output, and modulability (or lack of).

*We thank the reviewer for the suggestion. We included a new figure (Figure 1 in the revised manuscript) with the requested infographic, which will hopefully help to get an easier grasp of the model.*

2) I'm unsure that there is a well-established view of psychopathology as rigidity in meaning-making. Citations would help make your case. It's unlikely that psychopathology, in general, can be attributed simply to one element. Tenet 4 really ought to be toned down, it's completely over-generalizing as if this is a theory of everything.

*The issue about the p-factor as a single construct/element (or restricted set of latent elements/constructs) to which attribute psychopatology development, has more clearly been addressed and specific theoretical contributions have been listed in the revised manuscript.*

*In detail, the p-factor conceived as rigidity in meaning making has been introduced by two previous recent contributions (*De Luca Picione, 2020; Venuleo et al., 2020*), that have been specified in the text.*

*The core idea of Tenet 4 is in fact conceived as a general and superordinate respect to psychological constructs. In any case, it has been toned down. We hope it now sounds less hubristic: if this is the feeling the reviewer gets from the discussion, it was not our intention to induce this reaction.*

3) More talk about "meaning" would help as it is never defined in the paper. This paper, for example, <https://www.ncbi.nlm.nih.gov/pmc/articles/PMC3135310/> could be part of the introduction on the Harmonium model.

*We thank the reviewer for the suggestion of this very interesting article. We included it* (Tronick & Beeghly, 2011) *in a new paragraph presenting its perspective, hopefully managing to give a better representation of what is meant by “meaning”, especially from a developmental perspective.*

4) For the visual system, a fully-connected network does not represent a realistic architecture of the true brain's processing of an image. A convolutional neural network (CNN) is much more biologically plausible, which is likely why they also perform much better. I would do the same experiment on a CNN. Unsupervised training with Contrastive divergence and then using supervised learning of the final layer is rarely used nowadays. Modern visual neural networks do not work as such, and it is doubtful that human brains would work as such (although modern stochastic gradient descent (SGD) is still not entirely plausible either). I would start from a popular GitHub repository on a classification network, take a good CNN and its training algorithm rather than the very old-fashion architecture and algorithms you use.

*We agree with the Reviewer that modern machine vision systems are often based on different types of neural architectures, which include convolutional layers and possibly a variety of other processing constraints, such as inception layers, batch normalization or skip connections. However, as we now more clearly state in the manuscript, our goal here is not to implement a state-of-the-art computer vision system, but rather to operationalize a recent psychopathological theory in the form of a computational model. In cognitive modeling, simplification is a crucial step to improve interpretability* (McClelland, 2009)*, and this is also the reason why we opted for training and testing our model on a relatively simple dataset (MNIST) rather than on one of the large-scale datasets currently used in computer vision (e.g., ImageNet). Nevertheless, we now mention in the final discussion that an important step for future research would be to scale up our model to more realistic stimuli (e.g., faces and objects), which will likely require the implementation of more advanced machine vision architectures, such as those based on convolutional layers.*

*We also agree with the Reviewer that in AI and machine vision research it is common to use supervised feed-forward architectures. However, the unsupervised generative approach is one of the leading frameworks in cognitive modeling* (Testolin & Zorzi, 2016; Zorzi et al., 2013)*: energy-based models, such as deep belief networks trained with contrastive divergence* (Hinton, 2007)*, are considered very plausible from a cognitive and biological perspective because they can simulate a form of “learning by observation” that does not require labeled examples and exploits Hebbian learning principles* (O’Reilly, 1998)*. These models can also account for top-down effects in sensory processing* (McClelland et al., 2014)*, because learning corresponds to fitting an optimal probabilistic generative model to the sensory environment. Many computational principles underlying deep belief networks are empirically observed in the animal brain* (Fiser et al., 2010) *and are also well aligned with recent predictive coding theories of the cerebral cortex* (Clark, 2013; Friston, 2010) *at the same time providing a useful bridge with neurophysiological models based on spiking networks* (Pecevski et al., 2011)*. We further note that many recent cognitive models are indeed based on deep belief networks, covering a variety of cognitive domain ranging from numerical cognition* (Stoianov & Zorzi, 2012; Testolin, Dolfi, et al., 2020; Testolin, Zou, et al., 2020) *to letter perception* (Testolin et al., 2017)*, orthographic processing* (Di Bono & Zorzi, 2013; Testolin et al., 2016)*, shape perception* (Kubilius et al., 2016)*, emergence of object categories* (Sadeghi, 2016)*, development of object-action associations* (Grzyb et al., 2019) *and even the simulation of neurological syndromes* (Reichert et al., 2013)*. These models have also proven useful to simulate neurophysiological properties of single neurons in visual cortical areas* (Lee et al., 2008; Xiong et al., 2015)*. We do not think that our current manuscript is the right place to argue for or against the cognitive and biological plausibility of deep belief networks, but we hope to have clarified why we chose this neural network architecture to explore our computational theory of psychopathology.*

5) The whole experiment seems unnecessary because it is well known in modern AI literature that the inner layer's eigenspectrum is important. If one eigenvalue (or one principal component) dominates all others, then training is unstable, and results will be poor. Also, classifiers/discriminators used to trained Generative adversarial network (GAN) with poor eigenspectrum lead to the GAN only learning to generate one mode of the distribution (mode collapse).
You should review modern AI literature. I recommend looking at Charles H. Martin's work; see his blog: <https://calculatedcontent.com/>. He shows that the eigenspectrum of inner layers often fit a power-law (with parameter alpha), and low alpha is better (low alpha implies heavy tail distribution, so more entropy). Note that this work is on the networks' weights, not the inner's representation of your input. Nevertheless, both inner layer input and weights effectively answer a similar question (how collapsed/single-minded is your decision-making).

*We are grateful to the Reviewer for pointing out this interesting perspective. We agree that it is useful to put our findings in relation to the recent AI literature about the properties of inner layer’s eigenspectrum. We extended our manuscript in order to include and discuss recent relevant papers in the AI field* (Maddox et al., 2020; Martin et al., 2020; Martin & Mahoney, 2019)*. We nevertheless note (as also pointed out by the Reviewer) that these findings refer to the connectivity structure of the network rather than to its activations. Moreover, the novelty of our analysis also stems from the idea of analyzing how the dynamics (micro-stages) of the inner activations changes during the time course of learning, rather than looking at the inner eigenspectrum itself.*

6) We also know from the literature that a model trained on a low-variability dataset will not generalize well. Researchers often pre-train on imagenet (see VGG16) because it is a huge dataset, and then they fine-tune train on their small specific dataset. Result 1 from p13 is thus obvious.

*As we discuss in the manuscript, we acknowledge that in a machine learning setup low-variability datasets can result in overfitting, and we argue that from a behavioral standpoint this could share commonalities with rigidity in meaning-making. Again, our goal here was not to create a high-performance machine vision classifier (in that case one would start from a pre-trained network, as the Reviewer suggests), but rather to systematically manipulate the visual experience of the model and analyze the effects both on the behavior and on the model’s internal dynamics.*

7) About b in p13, there is no effect of perimetric complexity because the neural network has the capacity for learning much more complex tasks.

*We could not be sure about this a priori, because in similar models we did find an effect of perimetric complexity* (Testolin et al., 2017)*. Our previous findings showed that deep belief networks can exhibit effects of task complexity in their output responses; here we did not find an analogous phenomenon in our analysis of internal dynamics.*

8) "the network trained over the complex network" reword, p 13

*Done*

9) PC means two things; this is confusing (Principal components and perimetric complexity).

*Thank you. We replaced the acronym of perimetric complexity with PeCo.*

10) It seems arbitrary to consider wght_pd to be the first two factors and wght_sd to be all factors with eigenvalue less than 1. Looking at the distribution would be interesting.

*Given the novelty of the approach explored in this paper some degree of arbitrariness may be unavoidable, yet we tried our best to ground parameters choices on both theory and data. The different approach employed to choose the number of PC of WGHT_PD and WGHT_SD is due to the different meaning of these two indexes. On one hand, WGHT_PD represents those components that carry most of the weight in the classification process. The requirement of explaining 75% of variance is admittedly arbitrary, but it is also an arbitrary value that is customary in PCA literature, not unlike alpha = 0.05 in significance testing.
On the other hand, WGHT_SD was built to represent all residual components that were still relevant to the model, and here as well the choice of keeping eigen values greater than one was selected since it is the customary approach, and although we acknowledge that there are more sophisticated methodologies, there is still no consensus over which to use, and we feel that this article was already complex enough without introducing exoteric decision criteria. That being said, we included a new figure (figure 5 in the revised article) showing the distribution of explained variance and eigen values focusing on the two cut-off points.*

11) It's interesting to see that SD of wght_sd means better performance. It shows that the main PCs should stay stable, while minor ones should vary. But it does not really reflect adaptation to change and it's more just a process of learning. It could also reflect the eigenspectrum changing to more heavy-tail distributions.

*We agree that it is an effect of learning. Indeed, the idea of the harmonium mechanism we propose is that cognition is more efficient given sufficient “flexibility” in regard to secondary dimensions of meaning, and such flexibility is obtained through development in a more complex and richer environment. In this frame, we believe that in our toy model, the relationship between learning (higher WGHT_SD dispersion) and performance, represents a validation of our hypothesis.*

12) The connection between the theory and teh experiment is not convincing. It is far-reaching to connect the two, in my opinion.

*We thank the reviewer for highlighting this critical point. In revising the manuscript, particularly the changes to the introduction and "aims and hypothesis" section, we aimed to better clarify both the rationale of study and the reasons behind the chosen methodology. We hope that the new version of the manuscript resolves this concern.*

13) I would call it a "simulation study", not a "study".

*This suggested edit has been included in the Title of the manuscript*

14) The following would be needed in the rewriting of this paper:

- make it more approachable for a broad audience; right now, very few people in the world will understand the content
- use of modern deep learning simple tools (VGG or ResNet in TensorFlow or Torch) for your experiments.
- use of the experiment as an extra, not as the main course. Moreover, use multiple seeds to rerun your experiments since neural networks can be very unstable.
- focus more on bringing AI literature on eigenspectrum and the like to the argument and discussion as your main course

*We thank the Reviewer for these useful suggestions. Please note that in our simulations for each network condition (TC-HE and TC-LE) we repeated the unsupervised training procedure for 20 times, each time using a different randomization seed, in order to check the robustness of our analyses. Overall, we agree that it is important to bring AI literature closer to the cognitive modeling literature (and to the psychopathology modeling literature, in our particular case). We hope that our revised manuscript will be a first step in this direction, and we are looking forward to further extend this connection in future studies. We also think that the current level of discussion would benefit the general audience of Plos ONE, which is indeed very much multidisciplinary.*

15) reframe how these simulations provide arguments in favor of the Harmonium model being the correct one, ie frame it as using AI theory and simulations to understand better and test your Harmonium model. This is not proof of the Harmonium model being general psychopathology. There are significant gaps between humans brain functioning and artificial neural networks. The connection as it stands is very weak.

*A frame of how AI simulations can provide arguments in favor of the Harmonium model has been included in the discussion section.*

**REFERENCES MENTIONED IN REVIEWERS RESPONSES LETTER** (the great majority of them have been also added in the revised version of the manuscript)

Clark, A. (2013). Whatever next? Predictive brains, situated agents, and the future of cognitive science. *Behavioral and Brain Sciences*, *36*(3), 181–204. https://doi.org/10.1017/S0140525X12000477

De Luca Picione, R. (2020). Models of semiotic borders in psychology and their implications: From rigidity of separation to topological dynamics of connectivity. *Theory & Psychology*, 095935432094718. https://doi.org/10.1177/0959354320947184

Di Bono, M. G., & Zorzi, M. (2013). Deep generative learning of location-invariant visual word recognition. *Frontiers in Psychology*, *4*(September), 635. https://doi.org/10.3389/fpsyg.2013.00635

Fiser, J., Berkes, P., Orbán, G., & Lengyel, M. (2010). Statistically optimal perception and learning: from behavior to neural representations. *Trends in Cognitive Sciences*, *14*(3), 119–130. https://doi.org/10.1016/j.tics.2010.01.003

Friston, K. (2010). The free-energy principle: A unified brain theory? *Nature Reviews Neuroscience*, *11*(2), 127–138. https://doi.org/10.1038/nrn2787

Grzyb, B. J., Nagai, Y., Asada, M., Cattani, A., Floccia, C., & Cangelosi, A. (2019). Children’s scale errors are a natural consequence of learning to associate objects with actions: A computational model. *Developmental Science*, *22*(4), 1–12. https://doi.org/10.1111/desc.12777

Hinton, G. E. (2007). Learning multiple layers of representation. *Trends in Cognitive Sciences*, *11*(10), 428–434. https://doi.org/10.1016/j.tics.2007.09.004

Kubilius, J., Bracci, S., & Op de Beeck, H. P. (2016). Deep Neural Networks as a Computational Model for Human Shape Sensitivity. *PLoS Computational Biology*, *12*(4), 1–26. https://doi.org/10.1371/journal.pcbi.1004896

Lee, H., Ekanadham, C., & Ng, A. Y. (2008). Sparse deep belief net models for visual area V2. *Advances in Neural Information Processing Systems*, *20*, 873--880.

Maddox, W. J., Benton, G., & Wilson, A. G. (2020). Rethinking Parameter Counting in Deep Models: Effective Dimensionality Revisited. *ArXiv*, *2003.02139*. http://arxiv.org/abs/2003.02139

Martin, C. H., & Mahoney, M. W. (2019). Traditional and Heavy Tailed Self Regularization in Neural Network Models. *Proceedings of the 36th International Conference on Machine Learning, in PMLR*, *97*, 4284–4293.

Martin, C. H., Peng, T., & Mahoney, M. W. (2020). Predicting trends in the quality of state-of-the-art neural networks without access to training or testing data. *ArXiv*, 1–22.

McClelland, J. L. (2009). The place of modeling in cognitive science. *Topics in Cognitive Science*, *1*(1), 11–38.

McClelland, J. L., Mirman, D., Bolger, D. J., & Khaitan, P. (2014). Interactive Activation and Mutual Constraint Satisfaction in Perception and Cognition. *Cognitive Science*, *38*, 1139–1189. https://doi.org/10.1111/cogs.12146

O’Reilly, R. C. (1998). Six principles for biologically based computational models of cortical cognition. *Trends in Cognitive Sciences*, *2*(11), 455–462. https://doi.org/10.1016/S1364-6613(98)01241-8

Pecevski, D., Buesing, L., & Maass, W. (2011). Probabilistic Inference in General Graphical Models through Sampling in Stochastic Networks of Spiking Neurons. *PLoS Computational Biology*, *7*(12), e1002294. https://doi.org/10.1371/journal.pcbi.1002294

Reichert, D. P., Series, P., & Storkey, A. (2013). Charles Bonnet syndrome: evidence for a generative model in the cortex? *PLoS Computational Biology*, *9*(7), e1003134. https://doi.org/10.1371/journal.pcbi.1003134

Sadeghi, Z. (2016). Deep Learning and Developmental Learning: Emergence of Fine-to-Coarse Conceptual Categories at Layers of Deep Belief Network. *Perception*, *45*(9), 1036–1045. https://doi.org/10.1177/0301006616651950

Stoianov, I., & Zorzi, M. (2012). Emergence of a “visual number sense” in hierarchical generative models. *Nature Neuroscience*, *15*(2), 194–196. https://doi.org/10.1038/nn.2996

Stojanov, D., Korf, J., De Jonge, P., & Popov, G. (2011). The possibility of evidence-based psychiatry: Depression as a case. In *Clinical Epigenetics* (Vol. 2, Issue 1, pp. 7–15). BioMed Central. https://doi.org/10.1007/s13148-010-0014-2

Stoyanov, D. (2020). The Reification of Diagnosis in Psychiatry. *Neurotoxicity Research*, *37*, 772–774. https://doi.org/10.1007/s12640-019-00139-2

Stoyanov, D., Kandilarova, S., Paunova, R., Barranco Garcia, J., Latypova, A., & Kherif, F. (2019). Cross-Validation of Functional MRI and Paranoid-Depressive Scale: Results From Multivariate Analysis. *Frontiers in Psychiatry*, *10*, 869. https://doi.org/10.3389/fpsyt.2019.00869

Testolin, A., Dolfi, S., Rochus, M., & Zorzi, M. (2020). Visual sense of number vs. sense of magnitude in humans and machines. *Scientific Reports*, *10*(10045). https://doi.org/10.1038/s41598-020-66838-5

Testolin, A., Stoianov, I. P., & Zorzi, M. (2017). Letter perception emerges from unsupervised deep learning and recycling of natural image features. *Nature Human Behaviour*, *1*(9), 657–664. https://doi.org/10.1038/s41562-017-0186-2

Testolin, A., Stoianov, I., Sperduti, A., & Zorzi, M. (2016). Learning orthographic structure with sequential generative neural networks. *Cognitive Science*, *40*(3), 579--606. https://doi.org/10.1111/cogs.12258

Testolin, A., & Zorzi, M. (2016). Probabilistic models and generative neural networks: Towards an unified framework for modeling normal and impaired neurocognitive functions. *Frontiers in Computational Neuroscience*, *10*(JULY). https://doi.org/10.3389/fncom.2016.00073

Testolin, A., Zou, W. Y., & McClelland, J. L. (2020). Numerosity discrimination in deep neural networks: Initial competence, developmental refinement and experience statistics. *Developmental Science*.

Tronick, E., & Beeghly, M. (2011). Infants’ meaning-making and the development of mental health problems. *American Psychologist*, *66*(2), 107–119. https://doi.org/10.1037/a0021631

Venuleo, C., Salvatore, G., Ruggieri, R. A., Marinaci, T., Cozzolino, M., & Salvatore, S. (2020). Steps towards a unified theory of psychopathology: The phase space of meaning model. *Clinical Neuropsychiatry*, *17*(4), 236–252. https://doi.org/10.36131/cnfioritieditore20200405

Xiong, H., Rodríguez-Sánchez, A. J., Szedmak, S., & Piater, J. (2015). Diversity priors for learning early visual features. *Frontiers in Computational Neuroscience*, *9*(August), 104. https://doi.org/10.3389/fncom.2015.00104

Zorzi, M., Testolin, A., & Stoianov, I. P. (2013). Modeling language and cognition with deep unsupervised learning: a tutorial overview. *Frontiers in Psychology*, *4*(AUG), 515. https://doi.org/10.3389/fpsyg.2013.00515
