# Supplementary material for: The phase space of meaning model of psychopathology: A computer simulation modelling study
Source: PLoS One. 2021 Apr 26;16(4):e0249320. doi: 10.1371/journal.pone.0249320 (PMC8075201; doi:10.1371/journal.pone.0249320)
Supplement: Attachment — Submitted filename: Response to Reviewers 2.docx [file pone.0249320.s002.docx]

Dear Professor Chi-Hua Chen,

Academic Editor of Plos One journal,

I am presently submitting a revision of the paper entitled, ‘The Phase Space of Meaning Model of Psychopathology: a Computer Simulation Modeling Study”, by Alberto Testolin, Johann R. Kleinbub Arianna Palmieri^,^ and me.

Since the reviewers did not raise any other further comment, beside the need for a language revision, we submitted the manuscript to a professional proofing service. We hope that this revised version of the manuscript will meet your standards for publication.

As requested by PLOS One Policy, a marked-up copy of the manuscript that highlights changes made to the original version labeled 'Revised Manuscript with Track Changes' has been uploaded. An unmarked version of the revised draft without tracked changes has been upload as a separate file labeled 'Manuscript'.

Best regards

Sergio Salvatore
