# Supplementary material for: The phase space of meaning model of psychopathology: A computer simulation modelling study
Source: PLoS One. 2021 Apr 26;16(4):e0249320. doi: 10.1371/journal.pone.0249320 (PMC8075201; doi:10.1371/journal.pone.0249320)
Supplement: Peer review history

# Peer review: Decision Letter 0

**DOI:** 10.1371/journal.pone.0249320.r001

**Authors:** Chi-Hua Chen

11 Dec 2020

PONE-D-20-34281

The Phase Space of Meaning Model of Psychopathology. An Initial Validation Through Computer Simulation Modeling

PLOS ONE

Dear Dr. Salvatore,

Thank you for submitting your manuscript to PLOS ONE. After careful consideration, we feel that it has merit but does not fully meet PLOS ONE’s publication criteria as it currently stands. Therefore, we invite you to submit a revised version of the manuscript that addresses the points raised during the review process.

Please submit your revised manuscript by Jan 17 2021 11:59PM. If you will need more time than this to complete your revisions, please reply to this message or contact the journal office at plosone@plos.org. When you're ready to submit your revision, log on to https://www.editorialmanager.com/pone/ and select the 'Submissions Needing Revision' folder to locate your manuscript file.

Please include the following items when submitting your revised manuscript:

- A rebuttal letter that responds to each point raised by the academic editor and reviewer(s). You should upload this letter as a separate file labeled 'Response to Reviewers'.
- A marked-up copy of your manuscript that highlights changes made to the original version. You should upload this as a separate file labeled 'Revised Manuscript with Track Changes'.
- An unmarked version of your revised paper without tracked changes. You should upload this as a separate file labeled 'Manuscript'.

If you would like to make changes to your financial disclosure, please include your updated statement in your cover letter. Guidelines for resubmitting your figure files are available below the reviewer comments at the end of this letter.

If applicable, we recommend that you deposit your laboratory protocols in protocols.io to enhance the reproducibility of your results. Protocols.io assigns your protocol its own identifier (DOI) so that it can be cited independently in the future. For instructions see: http://journals.plos.org/plosone/s/submission-guidelines#loc-laboratory-protocols

We look forward to receiving your revised manuscript.

Kind regards,

Chi-Hua Chen, Ph.D.

Academic Editor

PLOS ONE

Journal requirements:

When submitting your revision, we need you to address these additional requirements.

1. Please ensure that your manuscript meets PLOS ONE's style requirements, including those for file naming. The PLOS ONE style templates can be found at

https://journals.plos.org/plosone/s/file?id=wjVg/PLOSOne_formatting_sample_main_body.pdf and

https://journals.plos.org/plosone/s/file?id=ba62/PLOSOne_formatting_sample_title_authors_affiliations.pdf

2. In your Data Availability statement, you have not specified where the minimal data set underlying the results described in your manuscript can be found. PLOS defines a study's minimal data set as the underlying data used to reach the conclusions drawn in the manuscript and any additional data required to replicate the reported study findings in their entirety. All PLOS journals require that the minimal data set be made fully available. For more information about our data policy, please see http://journals.plos.org/plosone/s/data-availability.

Upon re-submitting your revised manuscript, please upload your study’s minimal underlying data set as either Supporting Information files or to a stable, public repository and include the relevant URLs, DOIs, or accession numbers within your revised cover letter. For a list of acceptable repositories, please see http://journals.plos.org/plosone/s/data-availability#loc-recommended-repositories. Any potentially identifying patient information must be fully anonymized.

Important: If there are ethical or legal restrictions to sharing your data publicly, please explain these restrictions in detail. Please see our guidelines for more information on what we consider unacceptable restrictions to publicly sharing data: http://journals.plos.org/plosone/s/data-availability#loc-unacceptable-data-access-restrictions. Note that it is not acceptable for the authors to be the sole named individuals responsible for ensuring data access.

We will update your Data Availability statement to reflect the information you provide in your cover letter.

[Note: HTML markup is below. Please do not edit.]

Reviewers' comments:

Reviewer's Responses to Questions

**Comments to the Author**

1. Is the manuscript technically sound, and do the data support the conclusions?

The manuscript must describe a technically sound piece of scientific research with data that supports the conclusions. Experiments must have been conducted rigorously, with appropriate controls, replication, and sample sizes. The conclusions must be drawn appropriately based on the data presented. 

Reviewer #1: Yes

Reviewer #2: No

**********

2. Has the statistical analysis been performed appropriately and rigorously? 

Reviewer #1: I Don't Know

Reviewer #2: No

**********

3. Have the authors made all data underlying the findings in their manuscript fully available?

The PLOS Data policy requires authors to make all data underlying the findings described in their manuscript fully available without restriction, with rare exception (please refer to the Data Availability Statement in the manuscript PDF file). The data should be provided as part of the manuscript or its supporting information, or deposited to a public repository. For example, in addition to summary statistics, the data points behind means, medians and variance measures should be available. If there are restrictions on publicly sharing data—e.g. participant privacy or use of data from a third party—those must be specified.

Reviewer #1: Yes

Reviewer #2: Yes

**********

4. Is the manuscript presented in an intelligible fashion and written in standard English?

PLOS ONE does not copyedit accepted manuscripts, so the language in submitted articles must be clear, correct, and unambiguous. Any typographical or grammatical errors should be corrected at revision, so please note any specific errors here.

Reviewer #1: Yes

Reviewer #2: Yes

**********

5. Review Comments to the Author

Please use the space provided to explain your answers to the questions above. You may also include additional comments for the author, including concerns about dual publication, research ethics, or publication ethics. (Please upload your review as an attachment if it exceeds 20,000 characters)

Reviewer #1: This paper examines in-depth the fundamental issue of the continuum between normality and psychopathology by use and implementation of computational models, including neural networks/computer simulation techniques. Authors manage to defend the view that there exists a dimensional model of psychopathology, anchored in the Phase Space of Meaning concept developed on the level of computational modelling. The paper will benefit from few amendments:

1. Deliver more precise and straigtforward conclusion in terms of core message to more general mental health audience without background in cognitive science. Perhaps a summary of main contributions would fit the purpose.

2. Include some critical premises from methodological perspective, which are relevant to that core message, e.g.

https://link.springer.com/content/pdf/10.1007/s13148-010-0014-2.pdf

https://www.frontiersin.org/articles/10.3389/fpsyt.2019.00869/full

Reviewer #2: The authors seek to prove that the Harmonium model can be used to explain general psychopathology. Their theory states that rigidity in meaning-making is psychopathology, which can be represented through the Phase Space of Meaning (PSM).

They hypothesize that a PSM that puts more emphasis on its primary dimensions (representing the global meaning of the environment exposed too) and rather than its secondary dimensions (representing minor subjective details of the environment) leads to psychopathology.

They test this by learning a classification network and looking at the network's inner layers through training. They conceptualize the change in primary dimensions as the standard deviation of the first two PCs (wght_pd) and the change in secondary dimensions as the standard deviation of the other PCs (wght_sd) (with eigenvalue higher than 1). They find that high SD for wght_sd means higher accuracy, while the opposite is true for wght_pd. Thus, they conclude that this reflects how focusing only on the primary dimensions leads to worse internal decisions (which means psychopathology from their theory).

The Harmonium model's description is quite complicated; I understand that compressing the description into a few pages is difficult. What would really help is some infographics representing the PSM, the ongoing signal, the output, and modulability (or lack of).

I'm unsure that there is a well-established view of psychopathology as rigidity in meaning-making. Citations would help make your case. It's unlikely that psychopathology, in general, can be attributed simply to one element. Tenet 4 really ought to be toned down, it's completely over-generalizing as if this is a theory of everything.

More talk about "meaning" would help as it is never defined in the paper. This paper, for example, https://www.ncbi.nlm.nih.gov/pmc/articles/PMC3135310/ could be part of the introduction on the Harmonium model.

For the visual system, a fully-connected network does not represent a realistic architecture of the true brain's processing of an image. A convolutional neural network (CNN) is much more biologically plausible, which is likely why they also perform much better. I would do the same experiment on a CNN. Unsupervised training with Contrastive divergence and then using supervised learning of the final layer is rarely used nowadays. Modern visual neural networks do not work as such, and it is doubtful that human brains would work as such (although modern stochastic gradient descent (SGD) is still not entirely plausible either).

I would start from a popular GitHub repository on a classification network, take a good CNN and its training algorithm rather than the very old-fashion architecture and algorithms you use.

The whole experiment seems unnecessary because it is well known in modern AI literature that the inner layer's eigenspectrum is important. If one eigenvalue (or one principal component) dominates all others, then training is unstable, and results will be poor. Also, classifiers/discriminators used to trained Generative adversarial network (GAN) with poor eigenspectrum lead to the GAN only learning to generate one mode of the distribution (mode collapse).

You should review modern AI literature. I recommend looking at Charles H. Martin's work; see his blog: https://calculatedcontent.com/. He shows that the eigenspectrum of inner layers often fit a power-law (with parameter alpha), and low alpha is better (low alpha implies heavy tail distribution, so more entropy). Note that this work is on the networks' weights, not the inner's representation of your input. Nevertheless, both inner layer input and weights effectively answer a similar question (how collapsed/single-minded is your decision-making).

We also know from the literature that a model trained on a low-variability dataset will not generalize well. Researchers often pre-train on imagenet (see VGG16) because it is a huge dataset, and then they fine-tune train on their small specific dataset. Result 1 from p13 is thus obvious.

About b in p13, there is no effect of perimetric complexity because the neural network has the capacity for learning much more complex tasks.

"the network trained over the complex network" reword, p 13

PC means two things; this is confusing (Principal components and perimetric complexity).

It seems arbitrary to consider wght_pd to be the first two factors and wght_sd to be all factors with eigenvalue less than 1. Looking at the distribution would be interesting.

It's interesting to see that SD of wght_sd means better performance. It shows that the main PCs should stay stable, while minor ones should vary. But it does not really reflect adaptation to change and it's more just a process of learning. It could also reflect the eigenspectrum changing to more heavy-tail distributions.

The connection between the theory and teh experiment is not convincing. It is far-reaching to connect the two, in my opinion.

I would call it a "simulation study", not a "study".

The following would be needed in the rewriting of this paper:

- make it more approachable for a broad audience; right now, very few people in the world will understand the content

- use of modern deep learning simple tools (VGG or ResNet in TensorFlow or Torch) for your experiments.

- use of the experiment as an extra, not as the main course. Moreover, use multiple seeds to rerun your experiments since neural networks can be very unstable.

- focus more on bringing AI literature on eigenspectrum and the like to the argument and discussion as your main course

- reframe how these simulations provide arguments in favor of the Harmonium model being the correct one, ie frame it as using AI theory and simulations to understand better and test your Harmonium model. This is not proof of the Harmonium model being general psychopathology. There are significant gaps between humans brain functioning and artificial neural networks. The connection as it stands is very weak.

**********

6. PLOS authors have the option to publish the peer review history of their article (what does this mean?). If published, this will include your full peer review and any attached files.

If you choose “no”, your identity will remain anonymous but your review may still be made public.

**Do you want your identity to be public for this peer review?** For information about this choice, including consent withdrawal, please see our Privacy Policy.

Reviewer #1: No

Reviewer #2: No

[NOTE: If reviewer comments were submitted as an attachment file, they will be attached to this email and accessible via the submission site. Please log into your account, locate the manuscript record, and check for the action link "View Attachments". If this link does not appear, there are no attachment files.]

While revising your submission, please upload your figure files to the Preflight Analysis and Conversion Engine (PACE) digital diagnostic tool, https://pacev2.apexcovantage.com/. PACE helps ensure that figures meet PLOS requirements. To use PACE, you must first register as a user. Registration is free. Then, login and navigate to the UPLOAD tab, where you will find detailed instructions on how to use the tool. If you encounter any issues or have any questions when using PACE, please email PLOS at figures@plos.org. Please note that Supporting Information files do not need this step.

# Author comment: Author response to Decision Letter 0

**DOI:** 10.1371/journal.pone.0249320.r002

21 Jan 2021

please, see the file "response to Reviewers_Harmonium_NeuralNet_2021-01-17"

Attachment Submitted filename: response to Reviewers_Harmonium_NeuralNet_2021-01-17.docx Click here for additional data file.

# Peer review: Decision Letter 1

**DOI:** 10.1371/journal.pone.0249320.r003

**Authors:** Chi-Hua Chen

19 Feb 2021

PONE-D-20-34281R1

The Phase Space of Meaning Model of Psychopathology. A Computer Simulation Modeling Study

PLOS ONE

Dear Dr. Salvatore,

Thank you for submitting your manuscript to PLOS ONE. After careful consideration, we feel that it has merit but does not fully meet PLOS ONE’s publication criteria as it currently stands. Therefore, we invite you to submit a revised version of the manuscript that addresses the points raised during the review process.

Reviewer 1 suggested that authors are advised to provide additional round of proofreading prior to typesetting of the manuscript as it has some technical spelling errors. Please address reviewer's comments to revise and resubmit the manuscript.

Please submit your revised manuscript by Apr 05 2021 11:59PM. If you will need more time than this to complete your revisions, please reply to this message or contact the journal office at plosone@plos.org. When you're ready to submit your revision, log on to https://www.editorialmanager.com/pone/ and select the 'Submissions Needing Revision' folder to locate your manuscript file.

Please include the following items when submitting your revised manuscript:

- A rebuttal letter that responds to each point raised by the academic editor and reviewer(s). You should upload this letter as a separate file labeled 'Response to Reviewers'.
- A marked-up copy of your manuscript that highlights changes made to the original version. You should upload this as a separate file labeled 'Revised Manuscript with Track Changes'.
- An unmarked version of your revised paper without tracked changes. You should upload this as a separate file labeled 'Manuscript'.

If you would like to make changes to your financial disclosure, please include your updated statement in your cover letter. Guidelines for resubmitting your figure files are available below the reviewer comments at the end of this letter.

If applicable, we recommend that you deposit your laboratory protocols in protocols.io to enhance the reproducibility of your results. Protocols.io assigns your protocol its own identifier (DOI) so that it can be cited independently in the future. For instructions see: http://journals.plos.org/plosone/s/submission-guidelines#loc-laboratory-protocols

We look forward to receiving your revised manuscript.

Kind regards,

Chi-Hua Chen, Ph.D.

Academic Editor

PLOS ONE

[Note: HTML markup is below. Please do not edit.]

Reviewers' comments:

Reviewer's Responses to Questions

**Comments to the Author**

1. If the authors have adequately addressed your comments raised in a previous round of review and you feel that this manuscript is now acceptable for publication, you may indicate that here to bypass the “Comments to the Author” section, enter your conflict of interest statement in the “Confidential to Editor” section, and submit your "Accept" recommendation.

Reviewer #1: All comments have been addressed

**********

2. Is the manuscript technically sound, and do the data support the conclusions?

The manuscript must describe a technically sound piece of scientific research with data that supports the conclusions. Experiments must have been conducted rigorously, with appropriate controls, replication, and sample sizes. The conclusions must be drawn appropriately based on the data presented. 

Reviewer #1: Yes

**********

3. Has the statistical analysis been performed appropriately and rigorously? 

Reviewer #1: I Don't Know

**********

4. Have the authors made all data underlying the findings in their manuscript fully available?

The PLOS Data policy requires authors to make all data underlying the findings described in their manuscript fully available without restriction, with rare exception (please refer to the Data Availability Statement in the manuscript PDF file). The data should be provided as part of the manuscript or its supporting information, or deposited to a public repository. For example, in addition to summary statistics, the data points behind means, medians and variance measures should be available. If there are restrictions on publicly sharing data—e.g. participant privacy or use of data from a third party—those must be specified.

Reviewer #1: Yes

**********

5. Is the manuscript presented in an intelligible fashion and written in standard English?

PLOS ONE does not copyedit accepted manuscripts, so the language in submitted articles must be clear, correct, and unambiguous. Any typographical or grammatical errors should be corrected at revision, so please note any specific errors here.

Reviewer #1: Yes

**********

6. Review Comments to the Author

Please use the space provided to explain your answers to the questions above. You may also include additional comments for the author, including concerns about dual publication, research ethics, or publication ethics. (Please upload your review as an attachment if it exceeds 20,000 characters)

Reviewer #1: Authors are advised to provide additional round of proofreading prior to typesetting of the manuscript as it has some technical spelling errors.

**********

7. PLOS authors have the option to publish the peer review history of their article (what does this mean?). If published, this will include your full peer review and any attached files.

If you choose “no”, your identity will remain anonymous but your review may still be made public.

**Do you want your identity to be public for this peer review?** For information about this choice, including consent withdrawal, please see our Privacy Policy.

Reviewer #1: No

[NOTE: If reviewer comments were submitted as an attachment file, they will be attached to this email and accessible via the submission site. Please log into your account, locate the manuscript record, and check for the action link "View Attachments". If this link does not appear, there are no attachment files.]

While revising your submission, please upload your figure files to the Preflight Analysis and Conversion Engine (PACE) digital diagnostic tool, https://pacev2.apexcovantage.com/. PACE helps ensure that figures meet PLOS requirements. To use PACE, you must first register as a user. Registration is free. Then, login and navigate to the UPLOAD tab, where you will find detailed instructions on how to use the tool. If you encounter any issues or have any questions when using PACE, please email PLOS at figures@plos.org. Please note that Supporting Information files do not need this step.

# Author comment: Author response to Decision Letter 1

**DOI:** 10.1371/journal.pone.0249320.r004

12 Mar 2021

please, see the file "response to Reviewers"

Attachment Submitted filename: Response to Reviewers 2.docx Click here for additional data file.

# Peer review: Decision Letter 2

**DOI:** 10.1371/journal.pone.0249320.r005

**Authors:** Chi-Hua Chen

16 Mar 2021

The Phase Space of Meaning Model of Psychopathology. A Computer Simulation Modeling Study

PONE-D-20-34281R2

Dear Dr. Salvatore,

We’re pleased to inform you that your manuscript has been judged scientifically suitable for publication and will be formally accepted for publication once it meets all outstanding technical requirements.

Within one week, you’ll receive an e-mail detailing the required amendments. When these have been addressed, you’ll receive a formal acceptance letter and your manuscript will be scheduled for publication.

An invoice for payment will follow shortly after the formal acceptance. To ensure an efficient process, please log into Editorial Manager at http://www.editorialmanager.com/pone/, click the 'Update My Information' link at the top of the page, and double check that your user information is up-to-date. If you have any billing related questions, please contact our Author Billing department directly at authorbilling@plos.org.

If your institution or institutions have a press office, please notify them about your upcoming paper to help maximize its impact. If they’ll be preparing press materials, please inform our press team as soon as possible -- no later than 48 hours after receiving the formal acceptance. Your manuscript will remain under strict press embargo until 2 pm Eastern Time on the date of publication. For more information, please contact onepress@plos.org.

Kind regards,

Chi-Hua Chen, Ph.D.

Academic Editor

PLOS ONE

Additional Editor Comments (optional):

Reviewers' comments:

Reviewer's Responses to Questions

**Comments to the Author**

1. If the authors have adequately addressed your comments raised in a previous round of review and you feel that this manuscript is now acceptable for publication, you may indicate that here to bypass the “Comments to the Author” section, enter your conflict of interest statement in the “Confidential to Editor” section, and submit your "Accept" recommendation.

Reviewer #1: All comments have been addressed

**********

2. Is the manuscript technically sound, and do the data support the conclusions?

The manuscript must describe a technically sound piece of scientific research with data that supports the conclusions. Experiments must have been conducted rigorously, with appropriate controls, replication, and sample sizes. The conclusions must be drawn appropriately based on the data presented. 

Reviewer #1: Yes

**********

3. Has the statistical analysis been performed appropriately and rigorously? 

Reviewer #1: I Don't Know

**********

4. Have the authors made all data underlying the findings in their manuscript fully available?

The PLOS Data policy requires authors to make all data underlying the findings described in their manuscript fully available without restriction, with rare exception (please refer to the Data Availability Statement in the manuscript PDF file). The data should be provided as part of the manuscript or its supporting information, or deposited to a public repository. For example, in addition to summary statistics, the data points behind means, medians and variance measures should be available. If there are restrictions on publicly sharing data—e.g. participant privacy or use of data from a third party—those must be specified.

Reviewer #1: Yes

**********

5. Is the manuscript presented in an intelligible fashion and written in standard English?

PLOS ONE does not copyedit accepted manuscripts, so the language in submitted articles must be clear, correct, and unambiguous. Any typographical or grammatical errors should be corrected at revision, so please note any specific errors here.

Reviewer #1: Yes

**********

6. Review Comments to the Author

Please use the space provided to explain your answers to the questions above. You may also include additional comments for the author, including concerns about dual publication, research ethics, or publication ethics. (Please upload your review as an attachment if it exceeds 20,000 characters)

Reviewer #1: The manuscript is recommended for publication in its current form. It has addressed in a proper way the comments of the peer review.

**********

7. PLOS authors have the option to publish the peer review history of their article (what does this mean?). If published, this will include your full peer review and any attached files.

If you choose “no”, your identity will remain anonymous but your review may still be made public.

**Do you want your identity to be public for this peer review?** For information about this choice, including consent withdrawal, please see our Privacy Policy.

Reviewer #1: No

# Editor report: Acceptance letter

**DOI:** 10.1371/journal.pone.0249320.r006

**Authors:** Chi-Hua Chen

15 Apr 2021

PONE-D-20-34281R2 

The Phase Space of Meaning Model of Psychopathology: A Computer Simulation Modelling Study 

Dear Dr. Salvatore:

I'm pleased to inform you that your manuscript has been deemed suitable for publication in PLOS ONE. Congratulations! Your manuscript is now with our production department. 

If your institution or institutions have a press office, please let them know about your upcoming paper now to help maximize its impact. If they'll be preparing press materials, please inform our press team within the next 48 hours. Your manuscript will remain under strict press embargo until 2 pm Eastern Time on the date of publication. For more information please contact onepress@plos.org.

If we can help with anything else, please email us at plosone@plos.org. 

Thank you for submitting your work to PLOS ONE and supporting open access. 

Kind regards, 

PLOS ONE Editorial Office Staff

on behalf of

Professor Chi-Hua Chen 

Academic Editor

PLOS ONE
